# Rapid detection of methicillin-sensitive and resistanct Staphylococcus aureus and methicillin-resistant coagulase-negative Staphylococci from blood cultures by automated PCR assay

**DOI:** 10.1186/1753-6561-5-S6-P204

**Published:** 2011-06-29

**Authors:** JJ Hirvonen, S-S Kaukoranta

**Affiliations:** 1 Clinical Microbiology, Vaasa Central Hospital, Vaasa, Finland

## Introduction / objectives

We assessed the usefulness of a new automated molecular assay (GenomEra^TM^ MRSA Confirm, Abacus Diagnostica Oy, Finland), which amplifies simultaneously the marker of methicillin resistance (*mecA*) and a *Staphylococcus aureus*-specific DNA segment, in detection of methicillin sensitive and resistant *S. aureus* (MSSA and MRSA) and other methicillin resistant staphylococci (MRCoNS) in blood culture samples.

## Methods

We studied a total of 102 blood cultures (including 92 positive and 10 negative cultures) and two MRSA-positive control samples. All samples were inoculated into BacT/Alert enrichment bottles (bioMérieux, France) and analysed by routine diagnostic and antimicrobial susceptibility methods as well as GenomEra MRSA Confirm assay after a growth-positive signal or after a 7 days of incubation. The thermal cycling and detection were performed in GenomEra CDX instrument directly from diluted blood culture bottle medium without previous DNA extraction step. Results were obtained within an hour.

## Results

A total of 41 *S. aureus* (39 MSSA and 2 MRSA) and 27 MRCoNS strains were identified from the blood cultures and control samples byroutine methods. Of these, all except one were correctly identified by the GenomEra assay. The deviant result was obtained from a multi-bacterial sample, containing MRCoNS and methicillin susceptible staphylococcus other than *S. aureus* in a ratio of <1:1 000. Thus the overall sensitivity of the assay method was 98.5 %. For MRSA and MSSA, the sensitivity was 100 %. No false positive results were obtained with the assay, yielding a specificity of 100 %.

## Conclusion

GenomEra assay is a useful method for direct detection of MRSA, MSSA and MRCoNS in blood cultures. It offers a rapid support to infection control and clinical decision making.

## Disclosure of interest

None declared.

